# Transactivation and signaling functions of Tat are not correlated: biological and immunological characterization of HIV-1 subtype-C Tat protein

**DOI:** 10.1186/1742-4690-3-53

**Published:** 2006-08-18

**Authors:** Nagadenahalli Byrareddy Siddappa, Mohanram Venkatramanan, Prasanna Venkatesh, Mohanbabu Vijayamma Janki, Narayana Jayasuryan, Anita Desai, Vasanthapuram Ravi, Udaykumar Ranga

**Affiliations:** 1 Molecular Virology Laboratory, Molecular Biology and Genetics Unit, Jawaharlal Nehru Centre for Advanced Scientific Research, Bangalore, India; 2 Department of Neurovirology, National Institute of Mental Health and Neurosciences, Bangalore, India; 3 Microtest Innovations Pvt. Ltd, Bangalore, India

## Abstract

**Background:**

Of the diverse subtypes of Human Immunodeficiency Virus Type-1 (HIV-1), subtype-C strains cause a large majority of infections worldwide. The reasons for the global dominance of HIV-1 subtype-C infections are not completely understood. Tat, being critical for viral infectivity and pathogenesis, may differentially modulate pathogenic properties of the viral subtypes. Biochemical studies on Tat are hampered by the limitations of the current purification protocols. Tat purified using standard protocols often is competent for transactivation activity but defective for a variety of other biological functions. Keeping this limitation in view, we developed an efficient protein purification strategy for Tat.

**Results:**

Tat proteins obtained using the novel strategy described here were free of contaminants and retained biological functions as evaluated in a range of assays including the induction of cytokines, upregulation of chemokine coreceptor, transactivation of the viral promoter and rescue of a Tat-defective virus. Given the highly unstable nature of Tat, we evaluated the effect of the storage conditions on the biological function of Tat following purification. Tat stored in a lyophilized form retained complete biological activity regardless of the storage temperature. To understand if variations in the primary structure of Tat could influence the secondary structure of the protein and consequently its biological functions, we determined the CD spectra of subtype-C and -B Tat proteins. We demonstrate that subtype-C Tat may have a relatively higher ordered structure and be less flexible than subtype-B Tat. We show that subtype-C Tat as a protein, but not as a DNA expression vector, was consistently inferior to subtype-B Tat in a variety of biological assays. Furthermore, using ELISA, we evaluated the anti-Tat antibody titers in a large number of primary clinical samples (n = 200) collected from all four southern Indian states. Our analysis of the Indian populations demonstrated that Tat is non-immunodominant and that a large variation exists in the antigen-specific antibody titers.

**Conclusion:**

Our report not only describes a simple protein purification strategy for Tat but also demonstrates important structural and functional differences between subtype-B and -C Tat proteins. Furthermore, this is the first report of protein purification and characterization of subtype-C Tat.

## Background

Human Immunodeficiency Virus type-1 (HIV-1) exhibits high levels of genetic variation based on which the viral strains are classified into several distinct subtypes designated A through J [1]. Distribution of viral subtypes across the globe is non-uniform. Additionally, epidemic outbreaks due to recombinant forms of the viruses are also increasingly becoming a concern for global infections. Of the various subtypes, subtype-C has been successful in establishing rapidly growing epidemics in the most populous nations of Sub-Saharan Africa, Asia including India and China and Latin American countries like Brazil. Globally, subtype-C strains are responsible for nearly 56% of the infections [2]. The recent data emerging especially from southern Brazil [3] allude to proliferation proficiency of subtype-C viruses and such differences might partly be attributed to biological properties unique for this particular viral subtype. Although subtype-C viruses alone cause more infections than all other subtypes combined, relatively little is understood of their molecular and pathogenic properties. The current knowledge of HIV-1 pathogenesis is derived mostly from studies on subtype-B strains that have been prevalent in the US and Europe [4]. Whether the various genetic subtypes and recombinant forms of HIV-1 have biological differences with respect to transmission and disease progression, is controversial [5-8]. Tat, being critical for viral infectivity and pathogenesis, deserves attention with respect to differential pathogenic properties of the viral subtypes [9,10].

Tat, a key viral transactivator regulating gene expression from the viral promoter, is expressed early in the viral life cycle from the multiply spliced viral transcript [11]. Tat binds to the transactivation response element (TAR) that forms a stable RNA stem loop at the 5' end of all the viral transcripts and recruits pTEFb, consisting of Cyclin T1 and CDK9, to TAR. Hyper-phosphorylation of the carboxy terminal domain of RNA polymerase II by CDK9 leads to enhanced elongation of the transcription from the viral promoter [12,13]. In the presence of Tat, gene expression from the viral promoter is upregulated several hundred fold. In addition, Tat is secreted from productively infected cells into extracellular medium through a poorly defined pathway [14,15]. The extracellular Tat can reenter cells through the caveolar pathway [16] interacting with a variety of cellular receptors on the cell surface including heparan sulphate proteoglycans [17], and integrin receptors α5β1 and αvβ3 [18]. Extracellular Tat, readily taken up by cells, could reach the nucleus and modulate the expression of a variety of cellular genes including cytokines [19,20], chemokine coreceptors [21,22], MHC complex [23,24], and many others [25-27] thus contributing significantly to the overall pathogenicity of the virus.

Given the significant role Tat plays in regulating several critical viral and cellular properties and the important differences in the amino acid residues identified in the Tat proteins of diverse viral subtypes [9,28,29], a systematic evaluation of a possible correlation between Tat diversity and subtype properties is necessary [9,10]. Studies of this nature, however, require purification of large quantities of biologically functional Tat protein, which is relatively difficult to achieve using the existing methodologies. Protein purification of Tat from a recombinant source, especially *E. coli*, is wrought with several technical challenges mainly because of the intrinsic properties of this viral protein. Tat contains several functional domains each regulating multiple and often overlapping and complementary biological functions [30]. The presence of charged and hydrophobic amino acid residues in Tat makes protein purification difficult as this protein adheres to surfaces. Additionally, Tat containing a cysteine-rich domain, consisting of 6 or 7 cysteines, is prone to inactivation through oxidation and/or reduction. Tat also has an intrinsic tendency to multimerize and aggregate on storage and the multimer forms of Tat are biologically inactive [31].

Several protein purification strategies have been reported for recombinant Tat. One commonly used strategy is the expression of Tat with a peptide tag that would facilitate purification [31-34]. An alternative approach exploits Tat's natural affinity for heparin [15,35]. Most of the reported protein purification protocols have been standardized using Tat derived from a subtype-B source (B-Tat). Attempts to purify Tat from a subtype-C source (C-Tat), as well as subtype-B, using the reported protocols presented two problems. First, the yield was significantly poor and second, the protein lost its biological properties completely or partially. C-Tat is characterized by the presence of several signature amino acid residues [9]. Additionally, several of the standard protocols involve the use of a reverse-phase column chromatography for Tat purification [33,36-38]. In our hands, the bulk of Tat adhered to the hydrophobic columns and failed to elute from these columns resulting in poor protein yield. Moreover, protein eluted from such columns was often found to be biologically inactive, possibly a result of exposure to harsh organic solvents, suggesting that reverse-phase column chromatography might not be an ideal choice for the purification of a strongly hydrophobic protein like Tat. In view of these limitations, we developed a simple and efficient protein purification strategy for Tat that uses in tandem a Ni-NTA affinity column and an anion-exchange chromatography. We applied this purification strategy to C-Tat, as well as B-Tat, with high protein yield. A 6-amino acid His-tag was placed at the C-terminal end of the Tat proteins to permit Ni-NTA column purification. The protein was approximately 99% pure and biologically active as evaluated in a range of transactivation assays and signaling events. Here, we report not only a novel and simple protein purification strategy for Tat but also demonstrate that transactivation activity alone is not an optimal correlate for the functional integrity of Tat. Additionally, we demonstrate important differences between B- and C-Tat proteins at biological and structural levels.

## Results and discussion

### Purification and characterization of the recombinant Tat proteins

Although highly conserved within a viral subtype, the amino acid sequences of Tat are significantly diverse among viral subtypes. Such variation may underlie important biological differences of the viral subtypes. For instance, a recent study demonstrated natural variation at position 32 in subtype-E Tat, but not in subtypes B or C, causing selective inhibition of TNF gene transcription in Jurkat cells [10]. We previously demonstrated a natural substitution of a serine residue for a cysteine at position 31 and suggested attenuated chemokine function of subtype-C Tat for monocytes [9]. Given that the vast majority of the global viral infections are ascribed to subtype-C [2] and that Tat protein is a key viral factor significantly contributing to viral pathogenesis, we wanted to purify Tat from subtype-C origin for experimental evaluation. Our initial attempts at purifying Tat from bacterial lysates using reported protocols not only yielded low quantities of the protein but also the isolated protein was often completely or partially inactive.

**Figure 1:**
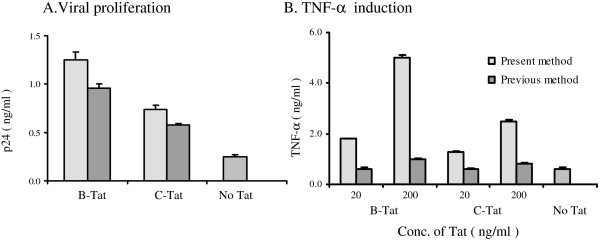
Discordance between the Transactivation and cytokine induction functions of Tat. (**A**) Rescue of the Tat-defective provirus. HLM-1 cells harbor an integrated provirus defective for Tat and produce large quantities of virus when complemented with a functional Tat protein. HLM-1 cells were incubated with B- or C-Tat (5 μg/ml) in complete medium or without Tat. Following Tat transfection, culture medium was collected at 24, 48 and 72 h and the levels of the viral structural protein, p24, secrerted into the medium was estimated using an antigen – capture assay. Data for the 72 h time point are presented here. Tat proteins (from subtypes -B and C) synthesized using a previously reporetd protocol were competent for the transactivation property. Tat proteins prepared using the protocol described in the present report were also transactivation competent (data not presented) (**B**) Induction of TNF-α secretion from primary monocytes. B- and C-Tat proteins prepared as above and as described in the present report were included for a comparative analysis. Monocytes freshly isolated from peripheral blood were incubated with the Tat proteins at two different concentrations (20 or 200 ng/ml) for 30 or 60 min. Cells were washed three times to remove Tat, resuspended in complete medium and incubated for 24 h. The amount of TNF-α secreted into the culture medium was assessed using a commericial kit following the manufacturer's instructions (R&D Systems). Tat proteins prepared using the protocol reported in the present manuscript successfully induced cytokine production from the monocytes in a dose-dependent manner. In contrast, Tat proteins prepared using reverse-phase chromatography failed in the induction. Note that the same Tat proteins were transactivation-competent and activated expression of a Tat-defective provirus (panel A above) and reporter genes under the viral LTR (data not presented).

Importantly, we repeatedly observed that the transactivation property of Tat was usually not affected by the protein purification strategy employed. The protein purification strategy used, however, dramatically affected many other biological functions of Tat often without modulating the transactivation property (Figure 1). Tat proteins purified from subtype-B and -C, using standard protocols that employ reverse-phase chromatography, successfully mediated production of a Tat-defective provirus from HLM-1 cells (Figure 1A) suggesting that the protein was capable of entering the cell and transactivating the viral promoter. These Tat proteins, however, failed to induce the secretion of TNF-α from monocytes (Figure 1B). In contrast, Tat proteins purified using the strategy reported here were competent for both transactivation and signaling properties (Figure 1B and see later).

**Figure 2:**
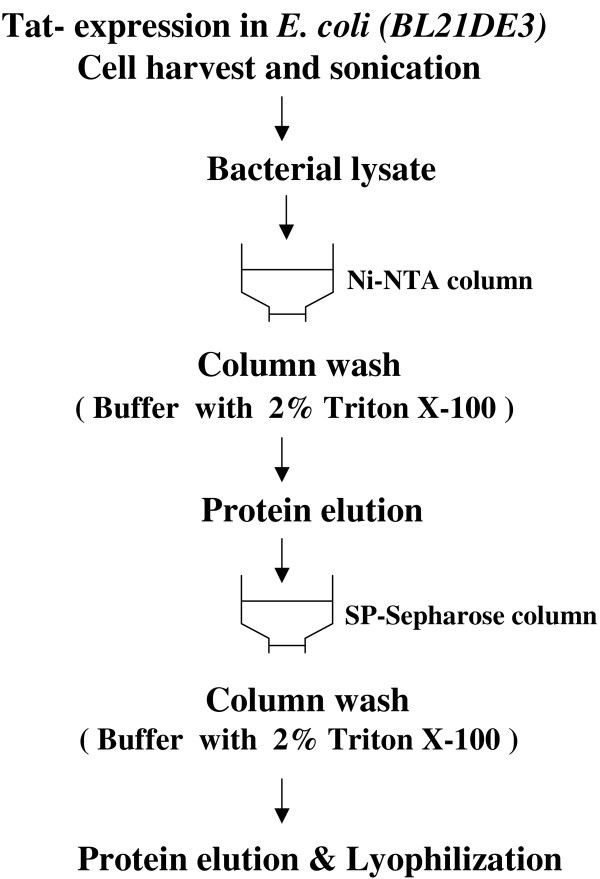
Schematic representation of isolation and purification of the Tat protein.

**Figure 3:**
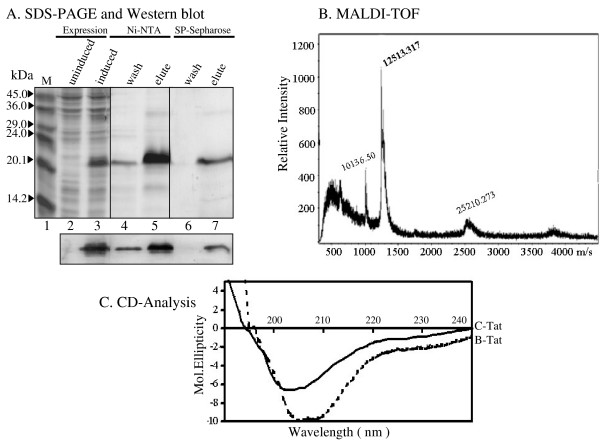
Purification and biochemical characterization of recombinantly expressed subtype-C Tat protein: (**A**) SDS-PAGE and Western blot analyses of the purified Tat protein. *E. coli *cells expressing Tat were harvested by centrifugation and lysed by sonication. Bacterial lysate was subjected to two successive strategies of protein purification, Ni-NTA and SP-Sepharose chromatographies. Measured quantity of the protein from different elutes was resolved on a 15% SDS-PAGE gel, M, protein molecular weight standards (# M3913, Sigma, St. Louis, Missouri, USA). Bottom pannel shows Western blot analysis of the Tat protein resolved on a duplicate SDS-PAGE gel and electrophoretically transferred to a PVDF membrane. A Tat-specific monoclonal antibody (# 4138, NIH AIDS Research and Reference Reagent Program) was used for the Western blot. (**B**) MALDI-TOF spectrum of the purified Tat protein. Purified and lyophilized Tat protein was reconstituted in sterile distilled water and subjected to MALDI-TOF analysis. (**C**) CD spectra of B-Tat and C-Tat were measured from 250 to 190 nm with a 0.1 cm path length in 10 mM phosphate buffer (pH 7.0).

In view of these limitations, we developed the present protein purification strategy for Tat without the use of the reverse-phase chromatography procedure. The protein purification strategy, as schematically depicted in Figure 2, consists of an affinity chromatography and an ion-exchange column chromatography, performed sequentially in that order. We amplified full-length Tat (101 residues) from an Indian subtype-C clinical sample, cloned it into a bacterial expression vector under the control of a T7 promoter and added a His-tag of 6 amino acid residues to the C-terminus of Tat to facilitate purification via Ni-NTA chromatography [39]. Tat protein eluted from the Ni-NTA columns was relatively pure and free of bulk of the bacterial proteins as assessed by SDS-PAGE electrophoresis (Figure 3A, lane 5). We found trace amounts of additional protein bands in this lane that are partly host proteins, partly Tat multimers (slow moving bands) and Tat degradation products (rapidly moving bands) as analyzed using Western blot (results not presented).

Tat protein eluted from Ni-NTA was directly applied to an anion-exchange column without removing imidazole. The isoelectric point (pI) of most of the Tat proteins is slightly alkaline (around pH 8 or above), whereas that of most of the *E. coli *host proteins is acidic, below pH 6 [40]. We took advantage of the difference in the pI values of Tat and *E. coli *host proteins and employed an anion-exchange chromatography in the purification strategy. At pH 6.8, where Tat was applied to the column, Tat was positively charged whereas *E. coli *proteins were expected to be negatively charged. Tat eluted from the Ni-NTA columns contained 300 mM Imidazole. Presence of imidazole at such a high concentration did not interfere with binding of Tat to the ion-exchange column. Tat eluted from the ion-exchange column is nearly free of bacterial proteins (Figure 3A, lane 7). Trace levels of additional protein bands above and below the main Tat band are multimers and degraded protein products of Tat, respectively, as seen in Western blot (data not presented). Thus, application of the Ni-NTA column chromatography and the ion-exchange chromatography, in tandem, not only efficiently removed *E. coli *proteins from Tat but also eliminated imidazole from the protein.

**Figure 4:**
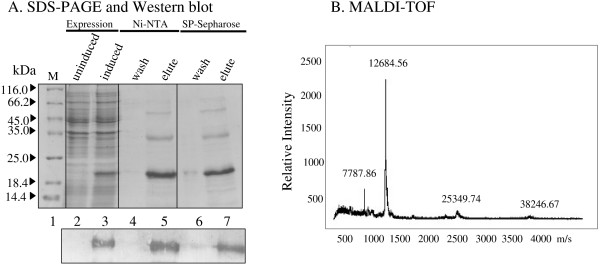
Purification and biochemical characterization of recombinantly expressed subtype-B Tat protein: (**A**) SDS-PAGE and Western blot analyses of the purified Tat protein. Tat expression cassette was amplified from a standard HIV-1 subtype B molecular clone, YU-2, and subcloned into a bacterial expression vector. *E. coli *cells expressing Tat were harvested by centrifugation and lysed by sonication. Bacterial lysate was subjected to two successive strategies of protein purification, Ni-NTA and SP-Sepharose chromatographies. Measured quantity of the protein was resolved on a 15% SDS-PAGE gel, M, protein molecular weight standards (# M3913, Sigma, St. Louis, Missouri, USA). Bottom pannel shows Western blot analysis of the Tat protein resolved on a duplicate SDS-PAGE gel and electrophoretically transferred to a PVDF membrane. A Tat-specific monoclonal antibody (# 4138, NIH AIDS Research and Reference Reagent Program) was used for the Western blot. (**B**) MALDI-TOF spectrum of the purified Tat protein. Purified and lyophilized Tat protein was reconstituted in sterile distilled water and subjected to MALDI-TOF analysis.

One serious limitation to recombinant protein expression in *E. coli *is the copurification of lipopolysaccharides (LPS) or endotoxin with the protein. LPS is the constituent component of Gram negative bacterial cell walls [41]. Endotoxins are negatively charged and copurify with similarly charged proteins like Tat. Even trace quantities of residual endotoxin could be highly toxic to the cells and tissues of mammalian origin especially those of human [42]. LPS could induce profound effects on mammalian cells, by engaging specific receptors on monocytes, macrophages and cells of other lineages and cause cell maturation, upregulation of costimulatory molecules, and release of several potent cytokines, prostaglandins, interleukins and platelet activating factors. Tat itself is known to induce secretion of several cytokines of immunologic significance from host cells [19,43,44]. Therefore, it is essential to remove endotoxin completely from Tat expressed from a bacterial source. We used Triton X-100 at 2% concentration to extensively wash the columns as Tat was immobilized on the columns. Above a critical concentration of the detergent, the lipid A component of endotoxins interacts with the micellar structures of the detergent and as a consequence of this interaction, endotoxins are efficiently separated from the aqueous phase [45,46]. Washing the columns with 2% Triton X-100, while Tat is bound on the columns, resulted in a reduction of endotoxin levels in the eluted Tat to the order of approximately 500 and 9,000 times after Ni-NTA and ion-exchange chromatographies, respectively (Table-1). The final concentration of endotoxin in the eluted Tat was approximately 0.04 EU/μg of protein which is within the acceptable limit [47]. The acceptable level of endotoxin for intravenous injections of plasmid DNA is 0.1 EU/μg [47] and 5 EU per KG weight of the recipient for recombinant proteins [46]. The presence of Tat was confirmed in Western blot using a Tat specific monoclonal antibody (Figure 3A) and the purity of the protein confirmed using MALDI-TOF (Figure 3B). The mass spectrum of C-Tat revealed a single major peak with a mass of 12513.317 as compared to the calculated mass of 12274.67 for a monomer. Tat degradation products were seen as minor low mass compounds and a Tat dimer at 25210.273. This analysis as well as the Western blot identified that the large proportion of Tat purified using the strategy reported here is a monomer with minor degradation products and a few multimers. We obtained essentially identical results with B-Tat purification. The SDS-PAGE, Western blot and MALDI-TOF profiles of B-Tat are presented in the figure 4.

**Table 1:** Recovery of Tat protein and removal of endotoxin

**Tat Sample**	**Endotoxin (EU/μg)**		**Yield (mg/Liter)**	
	
	**B-Tat**	**C-Tat**	**B-Tat**	**C-Tat**
Neat *E. coli* lysate	340	320	-	-
Ni-NTA (without Triton wash)	7.6	5.5	3.0	5.0
Ni-NTA (after Triton wash)	0.75	0.55	2.0	4.0
After SP-Sepharose	0.039	0.034	0.5	1.0

Tat protein recombinantly expressed in bacteria was purified using Ni-NTA column chromatography and ion-exchange column chromatography performed sequentially. Protein concentration of the eluted Tat protein was determined using a dye binding assay [99]. Endotoxin concentration in the samples was determined using a commercial kit (QCL-1000, Biowhittaker).

X-ray crystallographic information on Tat is lacking. Tat, like several other transactivation factors, is highly flexible and lacks well-structured three-dimensional folds [48,49]. Circular dichroism (CD) analyses of Tat derived from subtypes -B and -D in aqueous solutions identified primarily random coil structures and/or α-turns [50]. Importantly, minor structural variations in Tat are implied to influence the pathogenic properties of the viral protein [28]. Exon-1 of subtype-C Tat is characterized by a minimum of 6 signature amino acid residues [9]. To understand if amino acid variation between B- and C-Tat proteins could influence the secondary structure of the proteins and consequently their biological functions, we measured the CD spectra of C-Tat from 190 to 250 nm with a 0.1 cm path length in an aqueous buffer (Figure 3C). For comparison, Tat isolated from YU-2, a subtype-B molecular clone [51], was also included in the analysis. With B-Tat, as reported previously [52], we observed a negative band at 208 nM, typical of non-organized structures. Analysis of C-Tat, on the other hand, revealed two important differences. First, the negative band for C-Tat appeared at 204 nM, shifted from 208 nM of B-Tat. Second, the intensity of the negative band of C-Tat, as compared to B-Tat, was of lesser magnitude suggesting that C-Tat possibly might have a relatively higher ordered structure and be less flexible. The present study is the first report of the CD profile of subtype-C Tat protein. The results of the CD profile suggested that subtype-C Tat might be structurally different from subtype-B Tat. To understand the differences between C-Tat and other Tat proteins of other subtypes high resolution structures of nuclear magnetic resonance are needed.

### Recombinant Tat proteins are transactivation competent

Recombinantly expressed Tat is liable to inactivation by different mechanisms. Most of the previously published Tat purification protocols used the transactivation property of Tat alone to confirm functional integrity of Tat [15,31,53-55]. We, however, repeatedly observed that Tat protein purified using protocols published previously promoted expression of a reporter gene efficiently under the control of LTR or complemented replication of a Tat-defective provirus but failed to induce cytokine expression from primary monocytes or THP-1, a human acute monocytic leukemia cell line (Figure 1). Importantly, recombinant Tat protein obtained from reliable sources such as The NIH AIDS Research and Reference Reagent Program was also found to be defective in the non-transactivation properties of Tat (data not presented) underlining the importance of confirming the biological function of Tat using more than one property. Keeping this limitation in view, we simultaneously monitored two or more diverse and independent functions of Tat, the transactivation property, the cytokine induction and the coreceptor upregulation to confirm the functional integrity of the purified recombinant proteins.

**Figure 5:**
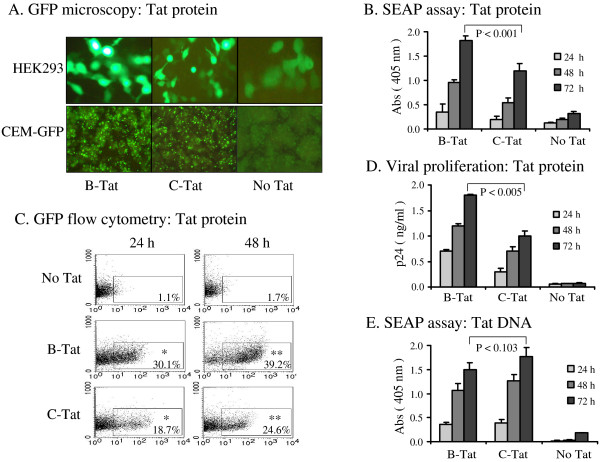
Evaluation of the transactivation property of the Tat proteins. (**A**) HEK293 cells (top panel) seeded in 12-well plates were transiently transfected with 0.5 μg of LTR-GFP reporter vector using a standard calcium phosphate protocol. Cells transfected with a blank plasmid were included as a negative control. LTR represents a full-length viral promotor cloned from an Indian primary subtype-C clinical isolate. Twenty four hours after the transfection, cells were incubated with freshly reconstituted Tat protein at a final concentration of 5 μg/ml in complete medium. Twenty four hours following the protein transfection, expression of GFP was documented using the UV-fluorescence microscopy. CEM-GFP cells (bottom panel), containing a stably integrated GFP gene under the control of subtype-B LTR, were transfected with 5 μg/ml of B or C-Tat proteins using a commercial lipid formulation following the directions of the manufacturer (Bioporter, Gene therapy systems, San Diego, CA, USA). or (**B**) HEK293 cells were transfected with a different reporter vector pLTR-SEAP and treated with Tat as described above. Expression of alkaline phosphate secreted into the medium was estimated at 24, 48 and 72 h using a colorimetric assay. The difference between B- and C-Tat treatments at all the time points was found to be statistically significant by Student's paired *t*-test. The *p *value at 72 h is shown. (**C**) CEM-GFP cells were treated with 5 μg/ml of B- or C-Tat proteins for the duration shown or left without treatment. Cells were harvested, fixed with 2% formaldehyde and evaluated for GFP expression using FACSCalibur flow cytometer (BD Biosciences). The live cells were gated on the basis of forward and side scatter. The number of GFP positive cells was determined by using scattergram of side scatter versus FL-1. Cells with fluorescence intensity greater than 10^1 ^were considered to be GFP positive and the gating was done accordingly. A total of 10,000 events were scored. The x-axis represents GFP intensity (FL-1) and the y-axis percentage of positive cells. Percent positive cells for the reporter protein are shown. The differences between B- and C-Tat treatments at both the time points were found to be statistically significant by Student's paired *t*-test. The *p *values at 24 and 48 h are < 0.0052* and < 0.0025**, respectively. (**D**) Rescue of a Tat-defective provirus. HLM-1 cells harboring an integrated provirus defective for Tat produce large quantities of virus when complemented with functional Tat protein. The cells were incubated with the Tat protein (5 μg/ml) in complete medium and 24, 48 and 72 h after Tat-transfection, the viral structural protein , p24, secreted into the culture medium was estimated using an antigen-capture assay. The difference between B- and C-Tat treatments at all the time points was found to be statistically significant by Student's paired *t*-test. The *p *value at 72 h is shown. (**E**) HEK293 cells were cotransfected with 0.5 μg of LTR-SEAP reporter vector and 0.1 μg of B-, C-Tat or empty vector. Alkaline phosphatase secreted into the medium was quantified at different time points as shown. All the above experiments were repeated several times and the data presented are representative of these experiments. CMV-β-galactosidase vector was used in all the transfections to control for differences in the transfection efficiency. β-galactosidase levels in the cell extracts were quantified using a colorimetric assay. All the quantitative assays were performed in triplicates and the data are presented as mean of triplicate values ± 1 S.D. The difference between B- and C-Tat treatments was not found to be statistically significant by Student's paired *t*-test. The *p *value at 72 h is shown.

Tat is believed to be secreted into extracellular spaces from infected cells, cross cell and nuclear membranes of the neighboring cells efficiently and transactivate latent viral promoter thus enhancing viral infectivity [14,56]. We tested the potential of the recombinant Tat proteins purified using the strategy reported here to enter the target cells and cause transactivation of the reporter genes under the control of the viral promoter. HEK293 cells were transiently transfected with a plasmid vector containing green fluorescent protein (GFP) under the control of the subtype-C LTR (C-LTR) and incubated for twenty four hours. Following the transfection, cells were incubated with B- or C-Tat proteins (5 μg/ml) for additional 24 hours and the expression of GFP was documented. Cells incubated with B- or C-Tat proteins expressed high quantities of GFP (Figure 5A, top panel). In contrast, cells transfected with the reporter vector but not exposed to Tat expressed low level GFP mainly as a result of Tat-independent transactivation from the LTR (Figure 5A). The reporter gene was placed under the regulatory control of a subtype-C LTR. Relatively high level basal transactivation was reported from C-LTR mainly as a consequence of subtype-specific differences in the regulatory motifs. Subtype-C LTR for instance contains one or more additional κB binding elements [57,58] as a result of which C-LTR is believed to be transcriptionally more active in the absence of Tat [59-61]. Regardless of the higher basal level gene expression, the GFP expression in HEK293 cells was significantly higher in the presence of Tat suggesting efficient Tat-transactivation by both the Tat proteins (Figure 5A). Higher levels of Tat-independent LTR transactivation, however, did not seem to be a problem when we used a different cell line CEM-GFP, a T-cell line stably transduced with a GFP reporter under the control of the HIV-1 LTR (Figure 5A, lower panel). We have analyzed and recorded GFP expression at several time points during the course of the experiment, typically at 24, 48 and 72 h. The data shown are from the 48 h time point. We observed identical reporter profiles at other time points.

To obtain quantitative information, we repeated the experiment with a vector expressing secreted alkaline phosphatase (SEAP) under the control of C-LTR. Culture supernatants were collected 24, 48 and 72 h after Tat incubation and levels of SEAP in the spent media were determined using a colorimetric assay. Both the Tat proteins induced expression of SEAP from the reporter vector in a time-dependent manner (Figure 5B). Interestingly, in both the reporter assays, B-Tat upregulated higher levels of protein expression as compared to C-Tat which was statistically significant by Student's paired t-test (*p *< 0.001). To confirm this observation, we transfected CEM-GFP cells with B- or C-Tat proteins and the GFP expression was evaluated by flow cytometry at 24 and 48 h following transfection. A significantly larger number of cells expressed GFP when transfected with B-Tat (30.1 and 39.2% at 24 and 48 h, respectively) as compared to C-Tat (18.7 and 24.6% at 24 and 48 h, respectively) while only a smaller fraction of the control cells were GFP positive (Figure 5C). The above experiments were repeated several times with different batches and different concentrations of the Tat proteins. B-Tat consistantly proved superior to C-Tat in these assays. Furthermore, we examined Tat-induced virion production from HLM-1 cells that contain a single copy of a Tat-defective provirus. Cells were incubated with 5 μg/ml of B- or C-Tat protein and the concentration of the viral antigen, p24, secreted into the medium was quantified using an antigen capture ELISA (Perkin Elmer Life Sciences, Boston, MA, USA). Both B- and C-Tat proteins entered the cells and complemented the defective provirus. Consistent with other assays, B-Tat released higher quantities of p24 into the medium at all the time points as compared to C-Tat and this difference was statistically significant, *p *< 0.005 (Figure 5D).

As mentioned above, we repeatedly noticed that C-Tat, as a protein, was found to induce relatively low level viral and reporter protein expression from both subtype-B and -C promoters in comparison with B-Tat (Figure 5A, B, C and 5D). In contrast, when delivered as a DNA expression vector (Figure 5E), C-Tat performed consistantly as efficiently as or even superior to B-Tat in transactivating the LTR (compare Figure 5B and 5E). The difference between B- and C-Tat DNA expression vectors was not found to be statistically significant (*p *= 0.103), the difference between Tat treatment and the controls, however, was found to be significant at all the time points. Carefully controlled experiments ruled out the possibility of differences in protein concentration, quality and conformation as a possible explanation for the observed differences between these two extracellular Tat proteins. It is possible that C-Tat is relatively less efficient than B-Tat in crossing cell membranes and we are presently evaluating this possibility. Our previous work identified several signature amino acid residues within C-Tat and such variations could differentially modulate biological properties of Tat [9]. Importantly, the arginine-glycine-aspartic acid (RGD) motif present in exon-2 is necessary for Tat to attach to the integrin receptors α5β1 and αvβ3 on the cell surface and enter the cells of diverse lineage including monocyte, T lymphocyte, vascular and skeletal muscle cells [62,63]. Tat binds these cells in a dose-dependent manner using the RGD motif and Tat mutants lacking the RGD motif fail to mediate efficient cell adhesion [62]. Interestingly, subtype-C Tat protein is naturally devoid of the RGD motif [1] and absence of this motif may adversely affect cell attachment of C-Tat and the subsequent internalization. We are presently comparing additional Tat clones from subtype-B and -C primary clinical isolates to confirm this observation.

### Recombinant Tat proteins mediate cytokine secretion and coreceptor upregulation

Extracellular Tat could contribute to viral pathogenesis by several different mechanisms [43,64-66] including activation of host cells to secrete cytokines and other immunologically potent molecules [44]. Cytokines secreted by host cells could further contribute to immune dysfunction manifested in AIDS [67]. Tat can drive transcription of a number of cytokine genes from cells of different lineage including monocytes, astrocytes, B-cells and T-cells [68]. Although the actual mechanism by which Tat induces cytokine secretion from the host cells is not completely understood, for some cytokines, engagement of specific receptor(s) on the cell surface appears to be necessary. Transient exposure of monocytes or astrocytes to Tat for periods as brief as 5 min is sufficient to induce secretion of cytokines from these cells for extended periods. Involvement of two different signaling pathways, the PKC pathway and the calcium pathway, could stimulate cytokine secretion from cells when exposed to Tat [69]. Additionally, exposure of Tat in the order of only milliseconds is sufficient to induce prolonged depolarization in neurons leading to neurotoxicity [70]. Collectively, these data suggest that transient and/or prolonged exposure of the host cells to Tat could result in a cascade of events leading to cell activation, cytokine secretion and immunomodulation.

**Figure 6:**
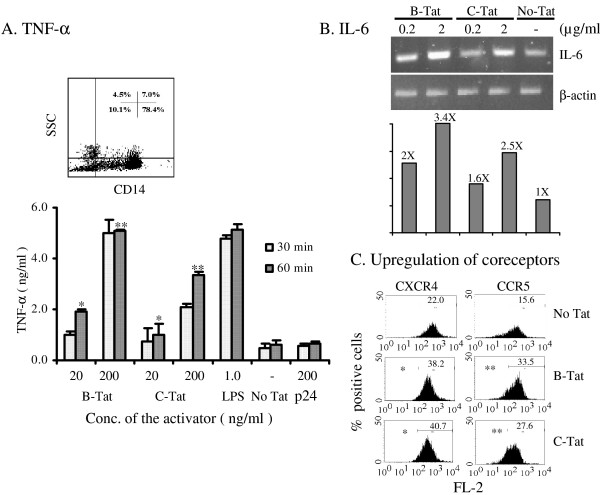
Activation of the cytokine signaling events by the Tat proteins. (**A**) Secretion of TNF-α from primary monocytes. Monocytes were isolated from peripheral blood by differential density gradient centrifugation and seeded in 96-well plates at 1 × 10^4 ^cells/well. The culture medium was supplemented with 20 or 200 ng/ml of B-or C-Tat proteins or left without Tat and incubated for 30 or 60 min. Cells were washed 3 times to remove Tat, resuspended in complete medium and incubated for 24 h. The level of TNF-α secreted into the culture medium was assessed using a commericial kit following the manufacturer's instructions. The experiment was repeated three times and the data presented are from one of the representative experiments. The data are presented as the mean value of triplicate wells ± 1 S. D. Inset, purity of the monocytes evaluated by flow cytometry. The forward vs. side scatter profile of cells isolated is presented. Approximately 86% of the gated cells are monocytes. The differences between B- and C-Tat treatments under all the conditions were found to be statistically significant by Student's paired *t*-test. For instance, at 60 min time point, the *p *values were < 0.05* for 20 ng and < 0.002** for 200 ng of Tat (**B**) Upregulation of the IL-6 transcript in U373-MAGI cells. U373 MAGI cells were exposed to B- or C-Tat at a concentration of 0.2 or 2 μg/ml or left without Tat treatment. Twenty four hours after Tat treatment, cells were lysed by directly adding Trizol to the wells and the total cellular RNA was isolated using standard protocols. An RT-PCR (5 ng input RNA) was performed to estimate the levels of IL-6 or β-actin transcripts. Amplified DNA fragments were resolved on a 1% agarose gel and visualized by ethidium bromide staining. The relative densities of the PCR fragments were determined by scanning the gel on a Phosphor Imager (FLA5000, Fuji). The intensity values of IL-6 fragments were normalized against β-actin and the fold upregulation in IL-6 transcript as a result of Tat treatment was presented in the bar diagram. (**C**) Tat-induced upregulation of chemokine receptors on monocytes. Monocytes were stimulated with 100 ng/ml B-, C-Tat proteins or cells were left without Tat treatment. Cells were harvested after 72 h, incubated for 20 min with PE conjugated monoclonal antibodies to human CXCR4 (12G5; Pharmingen), or CCR5 (2D7; Pharmingen), fixed with 2% formaldehyde and analyzed for coreceptor expression by FACSCalibur flow cytometer. The live cells were gated on the basis of forward and side scatter. Unstained monocytes were used as control. Expression of the chemokine receptors was analyzed using histograms with FL-2 on the x-axis and percent positive cells on the y-axis. A total of 10,000 events were scored. The differences between controls and Tat treatments were evaluated using Student's paired *t*-test and found to be statistically significant. In the case of CXCR4 upregulation, No Tat vs. B-Tat, *p *< 0.00004; No Tat vs. C-Tat, *p *< 0.00001; B-Tat vs. C-Tat, *p < 0.184. In the case of CCR5 upregulation, No Tat vs. B-Tat, *p *< 0.00001; No Tat vs. C-Tat, *p *< 0.001; B-Tat vs. C-Tat, ** *p *< 0.019.

Considering the importance of Tat-mediated cytokine induction for viral pathogenicity, we evaluated gene expression of two important cytokines, TNF-α and IL-6, from host cells exposed to recombinant B- or C-Tat proteins. Monocytes were isolated from fresh peripheral blood by two rounds of differential density gradient centrifugation [71]. A flow cytometric analysis of the monocytes using anti-human CD14 antibody conjugated to phycoerythrin (PE) identified these cells to be approximately 86% pure (inset Figure 6A). Monocytes were exposed to two different concentrations of Tat, 20 and 200 ng/ml, in complete medium for 30 or 60 min. Concentration of TNF-α was assessed in the spent media 24 h after Tat exposure using a commercial antigen capture assay (R&D Systems, Minneapolis, MN, USA). Both B- and C-Tat proteins induced high levels of cytokine expression from monocytes in a dose- and time-dependent manner (Figure 6A). LPS, at a concentration of 1.0 ng/ml, was used as a positive control for TNF-α secretion from monocytes. Interestingly, B-Tat, as in the transactivation assays, proved superior to C-Tat in inducing TNF-α secretion from the host cells although induction of cytokines is functionally a different assay and probably doesn't require cellular entry by Tat. To ensure that the trace levels of the endotoxin present in the protein preparations did not affect secretion of TNF-α from the cells, we included an additional control of the HIV-1 structural protein p24. p24 was isolated essentially using the same protein purification strategy as for Tat and contained low levels of endotoxin, below the detection limit. The viral structural protein failed to induce TNF-α from the target cells suggesting that the differential cytokine induction by the two Tat proteins is probably the result of the intrinsic differences between these two Tat proteins. Additionally, Tat proteins boiled for 30 min failed to induce TNF-α although endotoxins are heat stable (data not presented). Importantly, the protein solutions (B-Tat, C-Tat and p24) contained trace levels of endotoxin, below 0.05 EU/μg of protein that corresponds to 5 pg of endotoxin. Such low concentration of endotoxin must be too inadequate to be of functional significance for cytokine induction or other biological properties. Since the flow cytometric analysis of the monocyte preparation indicated the presence of cells of non-monocyte origin, we repeated these experiments using human monocyte (THP-1) and astrocyte (U373MG) cell lines. We obtained identical results with THP-1 cells, as with the primary monocytes, although overall magnitude of cytokine secretion was lower (data not shown). We also evaluated the induction of the IL-6 transcript in U373MG cells exposed to B- or C-Tat proteins, using a semi-quantitative RT-PCR. Expression of IL-6 mRNA was upregulated 2.0 and 3.4 fold with B-Tat and 1.6 and 2.5 fold with C-Tat when the cells were treated with 0.2 and 2.0 μg/ml of the corresponding Tat protein, respectively, as compared to the control cells (Figure 6B). Furthermore, we evaluated the ability of the Tat proteins to upregulate coreceptor expression on the target cells. Tat was previously shown to upregulate expression of CXCR4 and CCR5 two fold or more on T-lymphocytes and monocytes and influence viral infectivity [21,22,72,73]. We exposed freshly isolated monocytes to 100 ng/ml of Tat for 72 h and evaluated coreceptor expression on these cells by flow cytometry. While primary monocytes expressed basal levels of CXCR4 and CCR5 in the absence of Tat, these levels nearly doubled when the monocytes were treated with B- or C-Tat proteins (Figure 6C). The difference in the surface expression levels of CCR5 or CXCR4 with and without Tat treatment was statistically significant as evaluated by Student's paired t-test (P < 0.0005).

The data obtained from primary monocytes and three different cell lines on the expression pattern of two different cytokines and chemokine coreceptors collectively proved that the recombinant Tat proteins isolated from two different viral subtypes are functionally active. We obtained similar data with the Tat proteins from three other viral subtypes A, D and E (data not presented). It is important to note that two different assays we used here to test the recombinant Tat, transactivation and cytokine induction, differ from each other with respect to the mode of Tat interaction with the cell. While in the transactivation assay, Tat must enter the cell and its nucleus to activate the viral promoter, for the activation of cytokine secretion a simple engagement of cell receptors on the membrane is probably sufficient. Recombinant Tat proteins prepared from two different viral subtypes (subtype -B and -C) using the protein purification strategy presented here were found functionally competent in activating the cell signaling as well as the transactivation events. They not only crossed cellular and nuclear membranes but also upregulated gene expression from viral promoter derived from two different viral subtypes (subtype-B and -C).

### Low magnitude anti-Tat humoral immune response in the sera of HIV-1 infected subjects

To confirm antigenic nature of the recombinant Tat, we analyzed humoral immune response in subtype-C infection. In the natural infection, Tat is poorly immunogenic partly because of its predominantly nuclear localization and partly because of its small molecular size. Humoral and cellular immune responses to this viral antigen are observed only in a small number of seropositive individuals and at a low magnitude when present [74-77]. Importantly, immune responses to Tat, humoral and cell-mediated immune responses, are inversely correlated to disease progression and appear to be protective [78-81]. Given the importance of antibody response to Tat, we evaluated the anti-Tat IgG antibody titers in a large number of primary clinical samples (n = 200) collected from all the four southern states of India using an indirect ELISA format. IgG antibodies directed against gp41 were also estimated in the same plasma samples using a commercial kit. Unlike Tat, gp41 is immuno-dominant and strong antibody responses are generated against a highly conserved epitope [82,83]. Detection of anti-gp41 antibodies in the HIV-seropositive samples serves as an internal standard to compare the quality of the humoral immune response against Tat on the one hand and helps confirm the seropositive status of the samples used in this study on the other hand. We also included a set of 150 plasma samples collected from healthy seronegative donors in this analysis.

**Figure 7:**
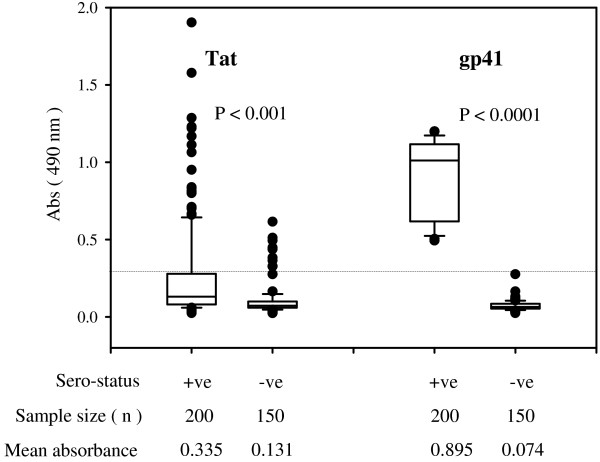
Seroreactivity of the Tat antigen. Determination of Tat and gp41-reactive IgG antibody titers in HIV-1 seropositive and control plasma samples. Plasma samples were diluted 1:100 and 1:50 for Tat and gp41 ELISAs, respectively. Each sample was tested in triplicate wells and the experiment was performed twice. n, the number of samples included in the study; *p *values and mean absorbance are shown in the box plots with the median represented by a horizontal line. The cut off value for the assay was indicated by a horizontal line.

Our analysis identified a large variation in the Tat-specific IgG antibody titers in the Indian plasma samples (Figure 7). The median antibody titer 0.335 of the HIV group was significantly higher than that of the control group with a median absorbance value of 0.131 (p < 0.001). Importantly, only a small number of the HIV plasma samples (18%, 36/200) contained Tat-reactive antibodies above an arbitrary cut off value of 0.3 absorbance. In contrast, 10 % (15/150) of the control sera exceeded this cut off value and overlapped significantly with the seropositive group. Seroreactivity of the control sera can be attributed to the existence of natural antibodies against Tat [84]. A large number of the HIV samples of southern India did not contain significant titers of Tat-reactive IgG antibodies confirming the previous reports from other populations demonstrating the non-immunodominant nature of Tat in the natural infection [74-77,85,86]. The present study is the first one to analyze anti-Tat IgG antibody titers in the Indian populations. Anti-gp41 IgG antibody response, unlike that of Tat, was not only of higher magnitude in these samples but also the titer variation was narrow confirming the immunodominant nature of this viral antigen. The median anti-gp41 antibody titer 0.895 was significantly higher than that of the control group 0.074 (p < 0.0001) confirming the seronegative status of the control population. Additionally, none of the control samples contained significant titers of antibodies to gp41. Additional analysis of the Tat-reactive antibodies identified an isotype class switch in all the HIV-1 seropositive plasma samples that fared above the cut off mark. Interestingly, isotype switching was not observed in normal sera with antibody titers above the cut off value (data not presented). We are presently evaluating the significance of the isotype switch to Tat neutralization and the relevance of Tat-reactive antibodies to disease progression in Indian clinical cohorts.

### Stability of the recombinant Tat protein under different conditions of storage

Tat, being a protein rich in highly charged and hydrophobic amino acid residues, is highly unstable and inappropriate storage conditions and handling could significantly affect its biological functions. The cysteine-rich domain of Tat contains 6 (subtype-C) or 7 (all other viral subtypes) highly conserved cysteines. Oxidation as well as reduction of Tat could lead to inactivation of the viral protein [49,87]. Additionally, Tat forms dimers and multimers and experimental evidence suggests that only the monomeric form of Tat is biologically active [31]. In view of the importance of storage and handling of Tat for preserving its biological functions, we evaluated and optimized these conditions.

**Figure 8:**
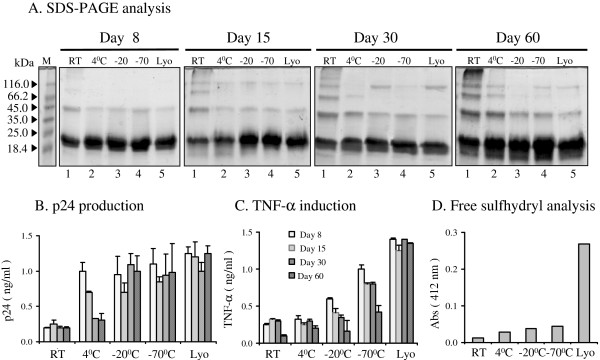
Infuence of the storage conditions on Tat functional stability. Freshly reconstituted Tat (in 20 mM Tris, pH 8, and 1 mM DTT) was divided into several aliquots and stored at different temperatures (RT, 4°C, -20°C, and -70°C) and incubated for different periods (0, 8, 15, 30 and 60 days). As a control, Tat was incubated at these different temperatures in a lyophilized form. (**A**) SDS-PAGE analysis. A portion of the aliquots was mixed with SDS-PAGE buffer, boiled and the samples were resolved on a 15% SDS-PAGE gel. M, protein molecular weight standards (# SM0431, MBI Fermentas); RT, room temperature. In parallel, portions of the same aliquots were used to evulate Tat's potential (**B**) to stimulate TNF-α production from primay monocytes (**C**) to rescue a defective provirus from HLM-1 cells and (**D**) to measure free sulfhydryl groups in Tat using DTNB assay. The experiment was performed twice and the data presented are from one representative experiment. Data are presented as mean value of triplicate wells ± 1 S.D.

After purification, recombinant Tat was lyophilized and stored in small aliquots at -70°C. To assess the stability of Tat, the lyophilized protein was reconstituted in a buffered solution and stored in the presence of 1 mM DTT, at different temperatures (ambient temperature, 4°C, -20°C, -70°C) for different time periods (8, 15, 30 and 60 days). Lyophilized Tat aliquots were also incubated along with the reconstituted protein samples for the same duration at the defined temperatures. Incubation of Tat for different periods was planned such a way that the harvest of the samples for the assay converged on the same day. At the end of the incubation, the physical configuration of Tat was examined using SDS-PAGE analysis. Additionally, biological activity of Tat was evaluated using two different assays, rescue of a Tat-defective virus (the transactivation property) and induction of TNF-α from the host cells (a cell signaling function). Multimer forms, dimers and higher forms, of Tat were seen in all the samples of Tat regardless of the storage condition and temperature (Figure 8A). Samples stored at room temperature and 4°C, formed excess quantities of multimer forms of the protein (compare lanes 1 and 2 across the panels, Figure 8A). These high molecular weight bands were visible by day 15 in the sample stored at the room temperature and on day 60, nearly half the total protein was identified in the multimerized form. In contrast, protein samples stored frozen (at -20°C and -70°C) appeared to be stable and like the freshly reconstituted sample, they did not form excessive multimer forms (Figure 8A). Although high mobility protein bands were seen in these samples, they constituted a relatively small fraction of the total protein.

Proteins stored at different temperatures for various periods were tested for their ability to rescue a Tat-defective provirus from the HLM-1 cells when these cells were incubated in the presence of Tat. Lyophilized Tat, regardless of the storage temperature, retained full transactivation activity, efficiently complemented Tat-defect in HLM-1 cells and induced production of large quantities of the virus from the cells (Figure 8B). Reconstituted protein stored frozen (at -20°C and -70°C) also retained transactivation activity for 60 days, although the lower temperature appears to be a better choice for storage. Tat stored at 4°C progressively lost the transactivation property as a function of time. This protein, however, retained a near complete activity up to day 8 and approximately 50% of the activity up to day 15. Tat stored at room temperature did not show any transactivation property at all. Overall there appears to be a direct correlation between the physical form of Tat (monomer vs. multimer) and its transactivation property.

We also simultaneously evaluated the influence of the storage conditions on the property of Tat to induce cytokine secretion from host cells. Lyophilized Tat, stored at different temperatures and freshly reconstituted in a buffer containing 1 mM DTT, regardless of the storage temperature, induced high quantities of TNF-α from human monocytes isolated from fresh blood (Figure 8C). Interestingly, the cytokine induction property of Tat, as opposed to the transactivation function of the same preparation, appeared to be more sensitive to the storage temperature of the protein solution. Tat stored even at -70°C rapidly lost its potential to induce TNF-α with time and did not contain significant biological activity after day 30. Tat stored at -20°C showed no significant activity after day 8, while Tat stored at room temperature or at 4°C completely lacked any potential to induce TNF-α at any time point. To understand if a correlation existed between Tat functional activity, especially cytokine induction, and the redox status of the Tat protein stored in a solution form for different periods, we used a modified Ellman reaction [88]. Ellman reagent 5,5-dithiobis (2-nitrobenzoic acid) reacts exclusively with free sulfhydryl groups in a dose-dependent manner and releases a yellow product 2-nitro-5-thiobenzoyate that is measured by colorimetry at 412 nM. Freshly reconstituted Tat was incubated for 60 days at different temperatures (room temperature, 4°C, -20°C, and -70°C) in the presence of 0.1 mM DTT. At the end of the incubation, a fresh vial of Tat was reconstituted and all the samples were assessed for free sulfhydryl groups in the Ellman reaction. We identified a direct correlation between the sulfhydryl content in the Tat solutions and their potential to induce cytokine production (Figure 8D). The lyophilized Tat and the Tat stored at room temperatures had the highest and lowest absorbance values at 412 nM (0.269 and 0.0134, respectively) with other Tat solutions having intermediary values between these extremes.

The cysteine-rich domain of Tat, containing 6 to 7 highly conserved cysteines, plays key role in transactivation, along with the core domain [89,90]. Except cysteine 31, all the other 6 cysteine residues are indispensable for the transactivation function [89,91]. A cooperative sharing of two metal ions among the 6 cysteines and one histidine of Tat and one cysteine of cyclin T1 is believed to be necessary for the transactivation function of Tat while these two proteins remain bound to TAR [92]. This model implies that all the 6 cysteines of Tat must be available in free thiol form to bind the metal ions, therefore these residues cannot form disulfide bonds. Experimental evidence, however, shows that significant proportion of recombinant Tat in vitro contains monomers that form disulfide bonds [93]. It is not known which of these two Tat monomers is biologically functional. For instance, Tat protein stored at 4°C for 8 days lacked both the transactivation (Figure 8B) and the cytokine induction functions (Figure 8C) although the monomeric form of Tat clearly constituted the bulk of the protein (Figure 8A). Based on these data, we believe that Tat monomer devoid of disulfide bonds is the biologically active form.

As discussed previously, cytokine secretion from cells, by and large, is induced by Tat via mechanisms different from that of transactivation. Tat is believed to engage certain receptors on the cell membrane and initiate a cascade of signaling events that ultimately results in the gene expression of target genes especially those of cytokines [15,17,63]. The activation of signal transduction is very rapid, often taking only a few minutes, as internalization of Tat into the cell probably is not necessary, a process that would require a few hours [94]. Importantly, the data we present here demonstrate that Tat depending on its functional integrity may be competent for one but not for a different kind of biological function. For instance, Tat protein stored at 4°C for 8 days was capable of rescuing the provirus from HLM-1 cells through its transactivation activity (Figure 8B) but the same preparation completely failed to induce TNF-α from monocytes (Figure 8C) although this sample did not form multimers at this time point and most of the protein remained as a monomer. The data presented here collectively demonstrate the need for stringent monitoring criteria for Tat biological functions. An enormously large number of biological functions has been ascribed to Tat [30]. Most often only the transactivation property of Tat is used to test the quality of Tat. We emphasize that at least one more biological function of the recombinantly produced Tat must be evaluated. Additionally, evaluation of the redox status of Tat, using a simple Ellman reaction could also testify to the functional competence of the Tat preparation.

## Conclusion

In summary, we reported a simple and efficient protein purification strategy for HIV-1 Tat. Using this protocol, we isolated highly purified Tat from subtypes B and C and demonstrated important differences in their structural properties. We have also standardized storage conditions for Tat and demonstrated the stability profile of Tat under diverse conditions of storage. Recombinant Tat was transactivation active. Furthermore, recombinant Tat successfully induced cellular signaling events, induced cytokine secretion and upregulated coreceptor expression on target cells. Using recombinant Tat, we identified antigen-specific antibodies in the Indian patient sera and showed that Tat is non-immunodominant in subtype-C natural infection. We also identified important functional differences between B- and C-Tat when they were delivered as protein to the target cell.

## Methods

### Cloning and expression of recombinant Tat proteins

The full-length C-Tat was cloned from a primary Indian clinical sample and B-Tat from YU-2, a molecular clone of subtype-B origin. For C-Tat cloning, peripheral blood was collected from a seropositive donor (BL43/02) and the mononuclear cells were isolated from the blood using the density gradient technique. Total RNA was extracted from the cells using the Trizol reagent (Sigma, St. Louis, Missouri, USA). Tat transcript was reverse transcribed using a sequence-specific reverse primer (N495: 5'-GCCCTCT**CTCGAG**GTCGAAGGGGTCTGTCTC-3'). The same primer in combination with a forward primer (N335: 5'-GAGGAG**CATATG**GAGCCAGTAGATCCTAAC-3') was used in the polymerase chain reaction to amplify the Tat gene. Restriction sites engineered into the primers, XhoI and NdeI, are bolded. The reverse transcription conditions were as follows: 30 min at 42°C and 5 min at 94°C. The amplification reaction mixture of 50 μl contained 250 nM of each primer, 100 μM each deoxynucleotide triphosphates, 1.25 U of *Taq *DNA polymerase, and 3.0 mM MgCl_2_. Amplification was performed using the following cycling conditions, pre-amplification denaturation for 30 s at 94°C, followed by 40 cycles, with each cycle consisting of incubations at 94°C for 30 sec, 60°C for 30 sec, and 72°C for 30 sec. The amplified product was purified using a column-based commercial kit (Qiagen, Hildel, Germany), and cloned directionally between NdeI and XhoI into a bacterial expression vector pET21b (Novagen, Madison, MI). As a result of cloning into this vector, a 6 amino acid His-tag was attached to Tat at the C-terminal. For subtype-B Tat cloning, HEK293 cells were transiently transfected with the pYU2 molecular clone (Cat # M2393, NIH AIDS Research and Reference Reagent Program) and incubated for 72 h. Extraction of the total RNA and cDNA preparation were as described above. The reverse primer (N529: 5'-CTCCACC**CTCGAG**ATGGACCGGATCTGTCTCTGT-3') was used in the reverse transcription reaction. This primer in combination with the forward primer N335 was used for amplification. The conditions for amplification and cloning were essentially as described above. The integrity of all the recombinant clones was confirmed by DNA sequencing. For the expression of recombinant Tat proteins, individual bacterial colonies (*E. coli *BL21(DE3)) were grown to 1 liter cultures in LB medium supplemented with 100 μg/ml ampicillin. Protein production was induced by adding IPTG to a final concentration of 1 mM to the cultures at 0.4 OD and incubating the cultures for additional 3 h. The bulk of the Tat protein was identified in a soluble form. Cells were harvested by high-speed centrifugation and resuspended in 20 ml of lysis buffer (20 mM Tris-HCl, pH 7.9; 10% glycerol; 0.4 mM, EDTA; 300 mM KCl; 0.1% IGEPAL; 10 mM imidazole; 0.2 mM PMSF and 1 mM DTT). Cells were lysed by sonication at 20 pulses at 3 min interval for 25 times (Branson Sonifier 450). The bacterial lysate was centrifuged at 16,000 rpm for 30 min at 4°C to remove cell debris. The lysate was stored frozen at -70°C until subsequent protein purification.

### Purification of the Tat protein

We used two different chromatography techniques, Nickel-NTA and SP-Sepharose, in tandem to purify Tat from bacterial cell lysate. The protein fraction eluted from the Ni-NTA columns was directly loaded to the SP-Sepharose column without delay and without removing imidazole. DTT at a concentration of 1 mM was included in all the reagents to minimize oxidation of Tat. All the materials that came in contact with Tat were siliconized to minimize protein loss by adherence to surfaces. Protein solutions were kept in dark to avoid photo-inactivation of Tat. The scheme for Tat purification is depicted in figure-2. A column (# 732-1010, BioRad, Hercules, CA) was packed with 2 ml of Ni-NTA beads (#70666, Novagen, Madison, MI) following the manufacturer's instructions and equilibrated with the lysis buffer. The bacterial cell lysate was repeatedly passed through the column at least four times to ensure protein binding. The column was washed thoroughly with 40 bed volumes of the wash buffer, (2% Triton X-100; 20 mM Tris-HCl, pH 7.9; 10% glycerol; 0.4 mM EDTA; 30 mM KCl; 0.1% IGEPAL; 50 mM imidazole; 0.2 mM PMSF and 1 mM DTT). Triton X-100, at 2% concentration, removed the bulk of endotoxin efficiently (Table-1). The washing procedure was repeated four additional times with the same buffer without Triton X-100. An additional wash with 0.5 M NaCl in phosphate buffer (pH 7.4) was used to remove proteins bound nonspecifically. Column-bound protein was eluted by passing 5 ml of the elution buffer (300 mM imidazole and 1 mM DTT in 50 mM phosphate buffer, pH 6.4). Eluted protein was collected in 1 ml aliquots for analysis or forwarded for the subsequent ion-exchange chromatography. While most of the *E. coli *proteins have a positive isoelectric point, HIV-1 Tat proteins are negatively charged (pI values of B- and C-Tat proteins used here are 9.56 and 8.80, respectively). We exploited the differences in the overall charge of Tat and the *E. coli *proteins and used an anion-exchange chromatography to remove traces of the host proteins from the preparations. One ml of the SP Sepharose fast flow Ion exchanger resin (#17-0729-10, Amersham Biosciences, Uppsala) was packaged into a column following the manufacturer's instructions. The column was equilibrated with 50 mM phosphate buffer. The protein eluted from the Ni-NTA column was directly applied to the SP Sepharose column. The column was washed four times with phosphate buffer containing 2% Triton X-100 and 1 mM DTT to remove any residual endotoxins that may have copurified in the first step of purification. Washing was repeated for four additional times with phosphate buffer without Triton X-100. Finally a single wash with 0.2 M NaCl in 20 mM Tris buffer (pH 8.0) was used to remove proteins bound nonspecifically. Bound Tat was eluted with 2.5 ml of elution buffer (0.5 M NaCl and 1 mM DTT in 20 mM Tris buffer, pH 8.0) and the eluted protein was collected in 5 aliquots of 0.5 ml each. The aliquots were immediately lyophilized and stored at -70°C until required.

### Western blot analysis of Tat

Recombinant Tat protein was boiled for 5 min in the gel loading buffer (250 mM Tris-HCl, pH 6.8, with 4% SDS, 20% glycerol, 0.01% bromophenol blue and 10% β-mercaptoethanol), resolved on a 15% SDS-PAGE gel and transferred to an Immobilon-P membrane (# IPVH00010, Millipore, Massachusetts). The membrane was blocked with 3% BSA and probed with a Tat-specific monoclonal antibody (# 4138, NIH AIDS Research and Reference Reagent Program), diluted 1:1,000 in PBS, at room temperature for 2 h. Membranes were washed and incubated with a secondary antibody conjugated to horseradish peroxidase. The blot was developed by incubating the membrane in the substrate solution containing 3 μl/ml H_2_O_2 _and 0.5 mg/ml 3,3'-diaminobenzidine tetrahydrochloride (Sigma) in PBS.

### Estimation of the endotoxin

Levels of endotoxin in the protein solutions were assessed using a commercial kit (QCL-1000, Biowhittaker, Walkersville, MD, USA) following manufacturer's instructions. Briefly, 50 μl of the samples or the endotoxin standards (a series of four two-fold dilutions) and blank containing endotoxin quality water were dispensed into duplicate wells of a microtiter plate pre-equilibrated at 37°C. To each well 50 μl of LAL reagent were added, contents mixed and plate incubated at 37°C for 10 min. This was followed by the addition of 100 μl of substrate solution to each well and additional incubation for 6 min at 37°C. The reaction was stopped by adding 100 μl of the stop reagent and the optical density was read at 405 nm. The concentration of endotoxin in the protein samples was evaluated by regression analysis.

### Mass spectrometry and circular dichroism measurments of C-Tat

Tat protein was dialyzed against water for 12 h and mixed with 1 μl of the matrix, prepared by adding 0.05% trifluoroacetic acid to a saturated solution of sinapinic acid (3,5-dimethoxy-4-hydroxycinnamic acid), and spotted onto the MALDI Plate. After the spot dried completely, it was excited with laser shots and the mass of the C-Tat was determined using MALDI mass spectrometry, on the Ultra Flex TOF/MALDI-TOF mass spectrometer (Bruker Daltonics, Billerica, MA). CD spectra were measured with 0.1 cm path length from 250-190 nm on a Jasco-810 spectropolarimeter. CD spectra were averaged for 2 or 3 accumulations. The samples were prepared in 10 mM phosphate buffer (pH 7.0) and the protein concentration was 225 μg/ml.

### DTNB assay for determining the free sulfhydryl content of C-Tat

10 ng of Tat protein incubated for 60 days at different temperatures (room temperature, 4°C, -20°C and -70°C) was mixed with 0.1 mM 5, 5'-dithiobis (2-nitrobenzoic acid) (DTNB) in 10 mM phosphate buffer at pH 8.0. After incubation for 20 min at room temperature, the absorbance at 412 nm was measured.

### Preparation of the human primary monocytes

Monocytes were isolated from fresh peripheral blood using a two-step density gradient strategy essentially as described previously [71]. Briefly, fresh blood was collected from healthy donors using vacuutainers containing EDTA (Becton Dickinson, Franklin). Blood was diluted by adding three volumes of PBS and overlayed above Ficol-Hypaque (1.077 g/ml, H8889, Sigma). The samples were centrifuged at 1,100 RPM for 30 min at room temperature. Peripheral blood mononuclear cells were retrieved from the interface and subjected to an additional round of density gradient centrifugation using percoll (1.064 g/ml,17-0891-01, Amersham Biosciences, Uppsala). The cells were centrifuged at 1,500 rpm for 30 min, recovered from the interface and washed 3 or 4 times with PBS. The monocytes were found to be approximately 86% pure by flow cytometry (Figure 5A, in set) using anti- human CD14- monoclonal antibody conjugated to PE (BD Pharmingen). The cell staining, fixing and flow cytometry protocols are described below. THP-1 and U373MG cells were maintained in RPMI and DMEM, respectively (Sigma) each supplemented with 10% fetal calf serum (Gibco-BRL, Gaithersburg, MD), penicillin (100 U/ml), streptomycin (100 mg/ml), and L- glutamine (2 mM).

### Transactivation assays for Tat

HEK293 cells were transfected with pLTR-GFP and treated with the Tat protein. Alternatively, HEK293 cells were cotransfected with the GFP reporter plasmid and a Tat-expression vector. CEM-GFP cells (# 3655, NIH AIDS Research and Reference Reagent Program) contain a GFP expression cassette under the control of subtype-B LTR. CEM-GFP cells were transfected with 5 μg/ml of Tat formulated in the Bioporter protein transfection reagent (Gene Therapy Systems, San Diego, USA). Twenty-four, 48 or 72 hours after the transfection, cells were harvested by scrapping (HEK293) or centrifugation (CEM-GFP) washed once with phosphate-buffered saline, resuspended in 500 μl of 1 mM EDTA in PBS, and fixed in formaldehyde at a final concentration of 2%. The cells were analyzed with a FACSCalibur flow cytometer (Becton Dickenson, San Jose, CA, USA) and the data analysis was performed using CellQuest Pro software. The live cells were gated on the basis of forward and side scatter. The number of GFP positive cells was determined by using scattergram of side scatter versus FL-1. Cells with fluorescence intensity greater than 10^1 ^were considered to be GFP positive and the gating was done accordingly. A total of 10, 000 events were scored. HLM-1 cells harboring an integrated virus defective for Tat produce large quantities of p24 if complemented for Tat defect. HLM-1 cells in 6-well culture plates were incubated with 5 μg/ml of recombinant Tat for 2 h in serum-free medium following which the medium was replaced by complete medium. Culture supernatant was sampled every 24 h. Empigen was added to each sample to a final concentration of 0.1%, and the samples were incubated at 56°C for 30 min to inactivate the virus and to release the p24 antigen. The quantity of p24 in the samples was measured using a commercial kit following manufacturer's instructions (Perkin Elmer life sciences, Boston, MA). Alkaline phosphatase secreted into the culture medium was quantified using a colorimetric assay as described previously [95]. In all transfections, we used CMV-β-galactosidase reporter vector as an internal standard for normalization. β-galactosidase activity in cell extracts was measured using a colorimetric assay [96].

### Evaluation of cytokine induction by Tat

Using primary monocytes, THP-1 and U373MG cell lines, we evaluated expression of two different cytokines, TNF-α and IL-6, after the cells were exposed to recombinant Tat proteins. Expression of TNF-α from monocytes was monitored 24 h after Tat exposure using a commercial kit (R&D Systems, Minneapolis, MN). Total RNA was isolated using Trizol reagent from U373MG cells with or without Tat exposure and first strand cDNA was prepared from total cellular RNA using random primers. PCR was performed using published primers for IL-6 and β-actin [97]. β-actin primers served as internal controls.

### Upregulation of chemokine-receptor expression on monocytes by Tat

Freshly isolated monocytes (5 × 10^5^) were stimulated with 100 ng/ml B- or C-Tat proteins or left without Tat treatment. Cells were harvested 72 h following Tat-treatment, resuspended in 100 μl of PBS, incubated for 20 min with PE conjugated monoclonal antibodies to human CXCR4 (12G5; Pharmingen, San Diego, California, USA) or CCR5 (2D7; Pharmingen). Cells were washed once with phosphate-buffered saline, resuspended in 500 μl of 1 mM EDTA in PBS, and fixed in formaldehyde at a final concentration of 2%. The cells were analyzed with a FACSCalibur flow cytometer (Becton Dickenson, San Jose, CA, USA) and the data analysis was performed using CellQuest Pro software. The live cells were gated on the basis of forward and side scatter. The flow cytometry data acquisition and analysis was carried out as mentioned above. Unstained monocytes were used as control and the expression of the chemokine receptors was analyzed using histograms with FL-2 on the x-axis and counts on the y-axis. A total of 10,000 events were scored.

### Enzyme-linked immunosorbent assay for Tat and gp41

A total of 200 peripheral blood samples were collected from HIV-seropositive volunteers (119 men, mean age 32.4 years, range 17–54; 40 women, mean age 29.76 years, range 20–56; 15 subjects below 15 years of age; in the rest details were not recorded). Study subjects, representing a heterogeneous community of social and demographic groups, were voluntary participants under the care of several government hospitals, private clinics, and referral centres dedicated to the service of HIV/AIDS, in the southern states of Karnataka, Tamil Nadu, Andhra Pradesh and Kerala. Most of the blood samples were collected over a period of 4 years (2001–2004), after informed consent, in EDTA vaccutainers (Beckton Dickinson, San Diego, CA, USA) from individuals who were identified to be HIV seropositive by multiple enzyme-linked immunosorbent assays and/or Western blots. The seronegative samples were collected from healthy donors from The Rotary Blood Bank, Bangalore. The Institutional Bioethics Committee of JNCASR and the Institutional Bioethics Committees at other participating institutions approved the study to evaluate humoral immune responses in the donor sera. The clinical profiles of all the subjects were described previously [98]. Plasma was separated by centrifugation and collected in 500 μl aliquots and stored frozen. Using an indirect ELISA format, we determined antigen-reactive IgG antibody titers to Tat and gp41 in the plasma samples collected from 200 seropositive and 150 seronegative donors. While the Tat-ELISA was developed in-house, a commercial kit (XCyton, Bangalore, India) was used for gp41 following the manufacturer's instructions. U-bottom micro titer wells (Greiner, Frickenhausen, Germany) were coated with 400 ng of Tat in 50 mM carbonate buffer (pH 9.6) and incubated at 37°C for 2 h. The wells were blocked with 3% BSA in PBS at 37°C for 1 h. Patient sera diluted 1:100 in 5% sheep serum containing 0.2% Tween 20 were added to appropriate wells and the strips incubated at 37°C for 1 h. The wells were washed five times with the wash buffer (50 mM Tris, pH 8.0; 100 mM NaCl; 0.2% Tween 20). Goat anti-human IgG antibody conjugated to Horse-radish peroxidase (Calbiochem, San Diego, CA) was diluted 1:10,000, added to each well and the strips were incubated at 37°C for 1 h. After thorough washing, 100 μl of substrate solution (0.1 M citrate monohydrate, 0.2 M disodium hydrogen phosphate, 1 mg/ml OPD and 0.3% hydrogen peroxide) was added to each well and the strips were incubated in dark at room temperature for 15 min. Enzyme reaction was stopped by adding 100 μl of 1 N HCl to each well. The absorbance was recorded at 495 nm using an ELISA reader (Molecular Devices, Sunnyvale, CA).

### Statistical methods

All the statistical analyses were performed with the SPSS package. Experiments were performed two or three times, and values obtained from three replicate samples were averaged in each experiment. Data are presented as mean value with the standard deviation (± 1 S. D.). Statistical significance was tested using Student's paired *t*-test. Differences were considered significant at *P *< 0.05.

## Abbreviations

B-Tat, Subtype-B Tat; CD, Circular Dichroism; C-Tat, Subtype-C Tat; ELISA, Enzyme-linked Immunosorbent Assay; GFP, Green Fluorescent Protein; HIV-1, Human Immunodeficiency Virus Type 1; LPS, Lipopolysaccharide; LTR, Long Terminal Repeat; PE, Phycoerythrin; SEAP, Secreted Alkaline Phosphatase

## Competing interests

The author(s) declare that they have no competing interests.

## Authors' contributions

NBS, MV, PV and MVJ performed the experimental work. NBS participated in drafting the manuscript. NJ participated in protein purification, experimental design and interpretation of the data. AD and VR involved in recruiting patients, collecting blood samples, experimental design and manuscript writing. UR conceived of the study, participated in its design and coordination and wrote the manuscript. All authors read and approved the final manuscript.
